# Low-Power Complementary Inverter Based on Graphene/Carbon-Nanotube and Graphene/MoS_2_ Barristors

**DOI:** 10.3390/nano12213820

**Published:** 2022-10-28

**Authors:** Dong-Ho Shin, Young Gyu You, Sung Il Jo, Goo-Hwan Jeong, Eleanor E. B. Campbell, Hyun-Jong Chung, Sung Ho Jhang

**Affiliations:** 1 School of Physics, Konkuk University, Seoul 05029, Korea; 2 Department of Advanced Materials Science and Engineering, Kangwon National University, Chuncheon 24341, Korea; 3 EaStCHEM, School of Chemistry, Edinburgh University, David Brewster Road, Edinburgh EH9 3FJ, UK; 4 Department of Physics, Ehwa Womans University, Seoul 03760, Korea

**Keywords:** complementary inverter, low power, graphene/carbon-nanotube junction, barristor

## Abstract

The recent report of a p-type graphene(Gr)/carbon-nanotube(CNT) barristor facilitates the application of graphene barristors in the fabrication of complementary logic devices. Here, a complementary inverter is presented that combines a p-type Gr/CNT barristor with a n-type Gr/MoS${}_{2}$ barristor, and its characteristics are reported. A sub-nW (~0.2 nW) low-power inverter is demonstrated with a moderate gain of 2.5 at an equivalent oxide thickness (EOT) of ~15 nm. Compared to inverters based on field-effect transistors, the sub-nW power consumption was achieved at a much larger EOT, which was attributed to the excellent switching characteristics of Gr barristors.

## 1. Introduction

Complementary metal oxide semiconductor (CMOS) devices have led to the development of science and technology using silicon. The density of integrated circuits (IC) gradually increased and the size of devices decreased, leading to Moore’s Law which showed that the integration of ICs doubled every two years. However, as silicon faced physical limitations for device scaling, new alternatives were needed [1]. Low-dimensional materials, such as nanotubes and layered materials are capable of channel dimension scaling and have excellent physical properties, presenting potential as new semiconductor materials [2,3,4]. In particular, graphene (Gr), which opened the research field of two-dimensional (2D) layered materials, has excellent electrical properties and its mobility can be up to ~100,000 cm${}^{2}$/Vs [5,6]. However, the absence of a band gap in graphene has limited its application in logic devices [7,8]. Transition metal dichalcogenides (TMDCs), such as MoS${}_{2}$ and WS${}_{2}$, have a band gap of about 1–2 eV. They have an indirect band gap in the bulk form and a direct band gap in the monolayer [9,10]. They have, therefore, been popular objects for study of their transport properties and incorporation into field-effect transistor (FET) structures [11,12,13,14]. However, the field-effect mobility was insufficient to be commercialized, and digital inverters based on TMDCs operated at high supply voltages causing high power consumption [15,16,17]. In addition, the majority of TMDCs were n-type, induced by structural defects and interfacial charge impurities. The lack of p-type 2D semiconductors also restricted the realization of CMOS logic function in 2D electronics [18].

In order to use graphene as the channel of a switching device, the graphene barristor, making use of the Schottky barrier at the junction between graphene and a semiconductor material, was invented [19]. The Schottky barrier formed in the junction between graphene and semiconductor, such as Si, TMDC, or organic semiconductor, etc., was modulated by changing the Fermi energy $E_{F}$ of graphene by applying a voltage to a gate electrode. Since the reverse current increased exponentially as the Schottky barrier height decreased, the graphene barristors exhibited excellent switching characteristics with a low subthreshold swing (SS) [19,20]. However, most Gr/TMDC barristors were also n-type because of the n-type nature of TMDCs, hindering the development of CMOS systems using Gr/TMDC barristors. Recently, by combining graphene with a semiconducting single-walled carbon nanotube (CNT), a p-type Gr/CNT barristor was demonstrated with an on–off current ratio of 10${}^{6}$ and a high mobility of μ~ 5350 cm${}^{2}$/Vs [21]. A low subthreshold swing of 70 mV/dec with an equivalent oxide thickness (EOT) of 15 nm was reported. In addition, high conductance of the CNT allowed Gr/CNT barristors to function at much lower supply voltages of 10–100 mV, making them promising candidates for low-power CMOS devices [21].

In this paper, a complementary inverter was fabricated using a p-type Gr/CNT barristor, combined with a n-type Gr/MoS${}_{2}$ barristor. The potential of the device as a low power complementary inverter was investigated and compared with previously reported low-power inverters based on low-dimensional materials.

## 2. Materials and Experimental

**Figure 1:**
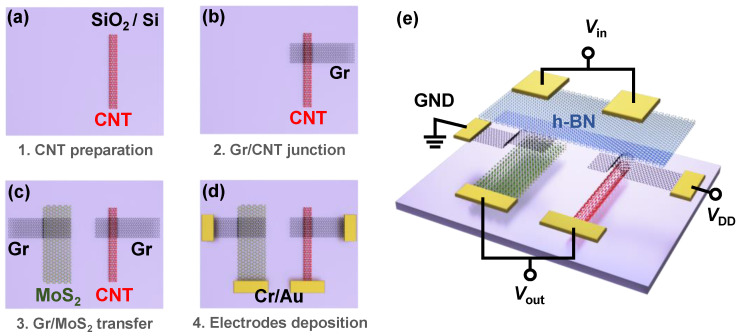
Fabrication processes of a complementary inverter based on Gr/CNT and Gr/MoS${}_{2}$ barristors. (**a**) A semiconducting CNT is transferred from a quartz to a SiO${}_{2}$(300 nm)/Si substrate. (**b**) A Gr/CNT junction is formed by the transfer of graphene onto the CNT. (**c**) A Gr/MoS${}_{2}$ junction, prepared on a different substrate, is moved next to the Gr/CNT junction. (**d**) Metal (Cr/Au) electrodes are patterned and deposited. (**e**) A large h-BN flake is transferred onto the junctions to be used as a top-gate insulator and top-gate electrodes are deposited.

Complementary inverters were fabricated based on Gr/CNT and Gr/MoS${}_{2}$ barristors. Single-walled carbon nanotubes (SWCNTs), straight and longer than 10 μm, were grown sparsely by chemical vapor deposition (CVD) on a quartz substrate employing ferritin as a catalyst [22,23]. Graphene, MoS${}_{2}$, and hexagonal boron nitride (h-BN) were prepared by mechanical exfoliation. Figure 1 illustrates the fabrication process of the complementary inverter using p-type Gr/CNT and n-type Gr/MoS${}_{2}$ Schottky junctions. The fabrication involved several transfer processes via the polymethylmethacrylate (PMMA) transfer method [24]. Firstly, SWCNTs were transferred from the quartz substrate to a SiO${}_{2}$(300 nm)/Si substrate and a CNT, isolated from other nanotubes, was identified using atomic force microscopy (AFM) and Raman spectroscopy (Figure 1a). The diameter $d_{t}$ of the SWCNTs was about 1.3 nm, determined from the Raman response of the radial breathing mode (RBM) by using $d_{t}=224$(cm${}^{-1})/\omega_{\mathrm{RBM}}$(cm${}^{-1}$) [25]. Graphene was then transferred onto the CNT to form a Gr/CNT junction (Figure 1b). On the other hand, a Gr/MoS${}_{2}$ junction was generated on a different SiO${}_{2}$/Si substrate in a similar fashion, and transferred adjacent to the Gr/CNT junction (Figure 1c). Conventional e-beam lithography was then used to create Cr(20 nm)/Au(20 nm) electrodes, as indicated in Figure 1d. Finally, a large 15 nm-thick h-BN flake was transferred covering both the Gr/CNT and the Gr/MoS${}_{2}$ junctions and used as gate dielectric, followed by the deposition of top-gate electrodes (Figure 1e). An AFM cleaning technique [26] was implemented to remove residual impurities on the material’s surface before each transfer process as the property of a barristor is very sensitive to the property of the junction.

For the operation of the complementary inverter, an input voltage $V_{\mathrm{in}}$ was applied through top-gates connected to each other. A positive $V_{\mathrm{DD}}$ was applied to the graphene constituting the Gr/CNT junction, and the other graphene comprising the Gr/MoS${}_{2}$ junction was grounded. This circuit connection allowed both barristors to be reverse-biased. An output voltage $V_{\mathrm{out}}$ was then measured at the electrodes connected to the CNT and MoS${}_{2}$, as shown in Figure 1e. Inverter characteristics were studied using a vacuum probe station and a Keithley 4200 semiconductor characterization system in Core Facility Center for Quantum Characterization/Analysis of Two-Dimensional Materials and Heterostructures.

## 3. Results

**Figure 2:**
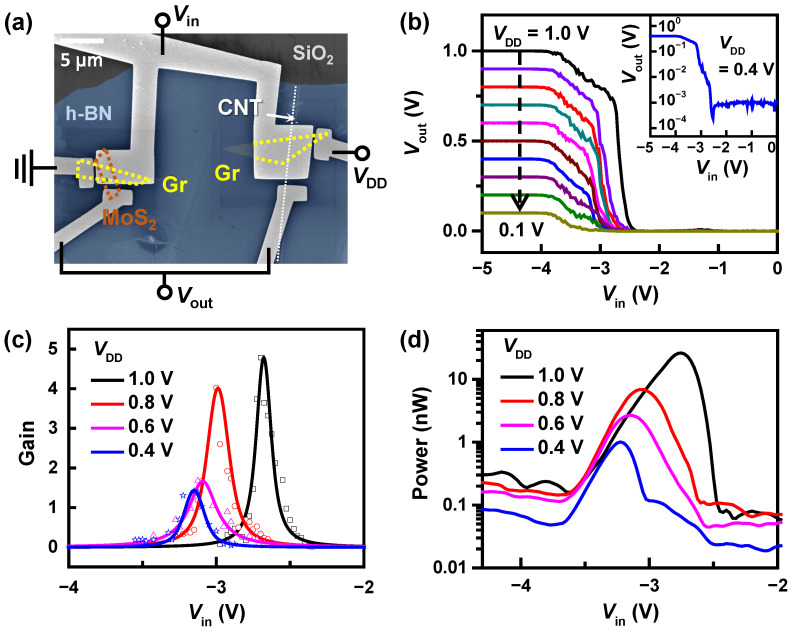
Inverter characteristics. (**a**) Scanning electron microscope image of Gr/MoS${}_{2}$ (left) and Gr/CNT (right) junctions used in a complementary inverter. Dotted lines indicate the location of 2D layers and CNT. Both junctions are covered by a 15 nm-thick h-BN flake. (**b**) Voltage transfer characteristics of the complementary inverter at different $V_{\mathrm{DD}}$. The inset shows the voltage transfer curve at $V_{\mathrm{DD}}=0.4$ V in a semilogarithmic scale. (**c**) Voltage gain as a function of the input voltage $V_{\mathrm{in}}$. (**d**) Power consumption of the complementary inverter versus $V_{\mathrm{in}}$ in a semilogarithmic scale.

Figure 2a shows a scanning electron microscope image of the Gr/MoS${}_{2}$ and Gr/CNT junctions used in a complementary inverter, where the thickness of the MoS${}_{2}$ was ~7 nm and the diameter of the semiconducting CNT was ~1.3 nm. A series connection of n-type Gr/MoS${}_{2}$ and p-type Gr/CNT barristors inverted the input signal $V_{\mathrm{in}}$ to its inverse $V_{\mathrm{out}}$. Figure 2b presents the voltage transfer characteristics (VTC) of the complementary inverter, obtained by varying $V_{\mathrm{DD}}$ from 1 V to 0.1 V at intervals of –0.1 V. A clear inversion of the input voltage was observed for the range of $V_{\mathrm{DD}}$ applied. When the input voltage was lower than –3.9 V, $V_{\mathrm{out}}≃V_{\mathrm{DD}}$, while, for $V_{\mathrm{in}}\geq -2.4$ V, the output voltage was inverted to $V_{\mathrm{out}}≃0$. The device operated under a negative input voltage as the threshold voltages of both p-and n-barristors had negative values, as shall be seen later. The inset of Figure 2b replots the VTC at $V_{\mathrm{DD}}=0.4$ V in a semilogarithmic scale. The ratio between the maximum and the minimum of $V_{\mathrm{out}}$ was $\sim 10^{3}$, and increased to $\sim 10^{4}$ at $V_{\mathrm{DD}}=1.0$ V, 100 times larger than that reported earlier for a complementary inverter based on Gr/Si barristors [19]. This implies high on/off current ratios of Gr/MoS${}_{2}$ and Gr/CNT barristors, and ensures a lower static power consumption at off-state. The threshold voltage of the inverter ($V_{M}$), where $V_{\mathrm{out}}=V_{\mathrm{DD}}/2$, was shifted to the left from –2.7 V to –3.6 V as $V_{\mathrm{DD}}$ decreased from 1 V to 0.1 V. The shift can be accounted for by the reverse bias-induced barrier lowering [27]. Figure 2c displays the voltage gain, defined as $\left|\frac{V_{\mathrm{out}}}{V_{\mathrm{in}}}\right|$, as a function of $V_{\mathrm{in}}$, shown for several $V_{\mathrm{DD}}$ between 0.4 and 1.0 V. The gain increased from 1.4 to 5 with the increase of $V_{\mathrm{DD}}$ from 0.4 V to 1.0 V. The gain further increased to ~12 at $V_{\mathrm{DD}}=1.6$ V (data not shown). A larger $V_{\mathrm{DD}}$ resulted in a larger gain due to the suppression of the contact barriers between semiconductors and metal electrodes at larger $V_{\mathrm{DD}}$. However, as the power consumed by the inverter increases with $V_{\mathrm{DD}}$, $V_{\mathrm{DD}}$ should be minimized and efforts have been made to lower the $V_{\mathrm{DD}}$ in logic devices [28]. The abruptness of the gain is indicated by the full width at half maximum (FWHM) being in the range of 120 and 230 mV. Figure 2d presents the power consumption of the inverter as a function of $V_{\mathrm{in}}$, extracted by the expression, $P=V_{\mathrm{DD}}\times I_{\mathrm{DD}}$. Here, $I_{\mathrm{DD}}$ is the current through the inverter. The power consumption was the largest between –3.9 V $<V_{\mathrm{in}}<$ –2.4 V, where a transition occurred in the magnitude of resistance between the p-type Gr/CNT and n-type Gr/MoS${}_{2}$ barristors, i.e., an indeterminate state. Consumed power is related to $V_{\mathrm{DD}}$, and the power consumption diminished with decreasing $V_{\mathrm{DD}}$. Although the static power consumption was negligible at ~0.1 nW, the peak power consumption was about 30 nW at $V_{\mathrm{DD}}=1$ V, and reduced down to ~1 nW at $V_{\mathrm{DD}}=0.4$ V. Optimization, such as the adjustment of the threshold voltages for each barristor and the reduction of EOT, can still improve the characteristics of the inverter, but the observed switching power of 1 nW at $V_{\mathrm{DD}}=0.4$ V is lower than, or comparable to previously reported low-power complementary inverters [29,30,31,32,33]. The low power operation is a consequence of the barristor characteristics. The barristor controls the current exponentially via modulation of the Schottky barrier height by changing the Fermi level of graphene. Thus, fast switching is possible even at a low voltage bias. The results before optimization already show that the complementary inverter based on p-type Gr/CNT and n-type Gr/MoS${}_{2}$ barristors possesses the potential as a low power inverter with a moderate gain, operating at a low $V_{\mathrm{DD}}$.

**Figure 3:**
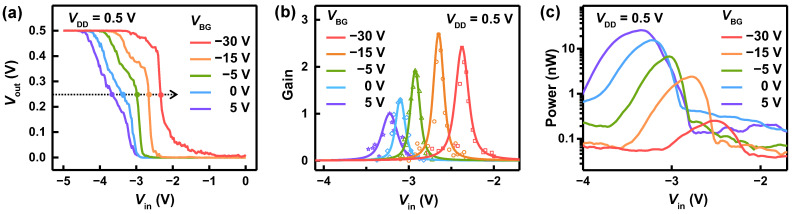
Inverter characteristics modulated with back-gate voltage $V_{\mathrm{BG}}$. (**a**) Voltage transfer characteristics of the complementary inverter at $V_{\mathrm{DD}}=$ 0.5 V, obtained by varying $V_{\mathrm{BG}}$. (**b**) Voltage gain as a function of the input voltage $V_{\mathrm{in}}$, measured at different $V_{\mathrm{BG}}$. (**c**) Power consumption of the inverter versus $V_{\mathrm{in}}$. Peak power is reduced down to 0.2 nW at $V_{\mathrm{BG}}=-$ 30 V.

By applying a back-gate voltage, $V_{\mathrm{BG}}$, through a SiO${}_{2}$(300 nm)/Si substrate, the performance of the inverter can be improved. Although the top-gate $V_{\mathrm{in}}$ tunes the work function of graphene, the back-gate voltage can modulate the work function of the semiconductors (CNT and MoS${}_{2}$) constituting the bottom of each junction on the substrate. Figure 3a exhibits VTC curves for $V_{\mathrm{DD}}=0.5$ V, measured at different $V_{\mathrm{BG}}$. As $V_{\mathrm{BG}}$ was decreased from 5 V to –30 V, i.e., in the direction of hole-doping to the CNT and MoS${}_{2}$, $V_{M}$ moved to the right from –3.7 V to –2.3 V, reflecting the shifts of threshold voltage for each barristor. Figure 3b shows how the voltage gain versus $V_{\mathrm{in}}$ changes with $V_{\mathrm{BG}}$. The voltage gain increased with decreasing $V_{\mathrm{BG}}$, providing a gain of 2.7 and 2.5 at $V_{\mathrm{BG}}=$ –15 V, –30 V, respectively. The FWHM of the gain was around 160 meV. Interestingly, as shown in Figure 3c, the power consumption of the complementary inverter was reduced by 100 times as $V_{\mathrm{BG}}$ was decreased from 5 V to –30 V. At $V_{\mathrm{BG}}=$ –30 V, the peak power consumed in the inverter was ~0.2 nW. Thus, a low-power (0.2 nW) complementary inverter with gain of 2.5, operating at a low $V_{\mathrm{DD}}=$ 0.5 V, was demonstrated with an EOT of ~15 nm, combining a p-type Gr/CNT barristor with a n-type Gr/MoS${}_{2}$ barristor.

**Figure 4:**
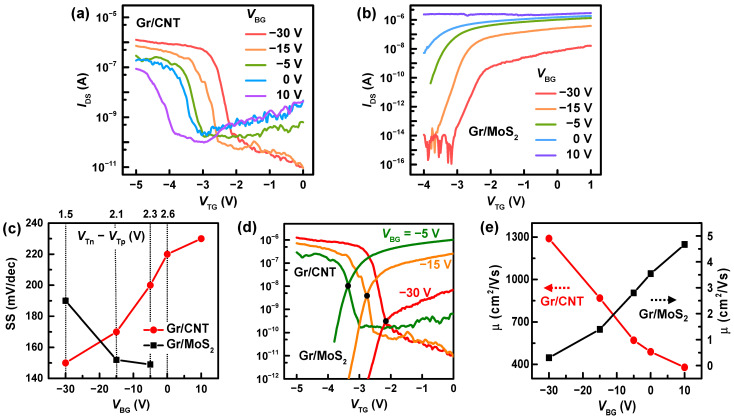
(**a**) Drain-source current, $I_{\mathrm{DS}}$, versus $V_{\mathrm{TG}}$ of the Gr/CNT barristor, obtained from various $V_{\mathrm{BG}}$. A reverse drain-source bias, $V_{\mathrm{DS}}$, of –0.5 V is applied to the CNT so that holes cross the Schottky barrier from graphene to CNT. (**b**) Transfer curves of the Gr/MoS${}_{2}$ barristor, measured at different $V_{\mathrm{BG}}$. $V_{\mathrm{DS}}=$ 0.5 V is applied to the MoS${}_{2}$, allowing electrons to cross the Schottky barrier from graphene to MoS${}_{2}$. (**c**) Subthreshold swing of the Gr/CNT and Gr/MoS${}_{2}$ barristors, deduced from transfer curves for different $V_{\mathrm{BG}}$. (**d**) Transfer curves of the Gr/CNT and the Gr/MoS${}_{2}$ barristors displayed together for selected $V_{\mathrm{BG}}$. The intersection of the transfer curves occurred at a lower current regime with decreasing $V_{\mathrm{BG}}$. (**e**) Estimated field-effect mobility as a function of $V_{\mathrm{BG}}$. See the left axis for the Gr/CNT barristor and the right axis for the Gr/MoS${}_{2}$ barristor.

The characteristics of the complementary inverter and the dependence on $V_{\mathrm{BG}}$ can be understood from the characteristics of individual barristors comprising the inverter. Figure 4a,b shows transfer characteristics of the p-type Gr/CNT and the n-type Gr/MoS${}_{2}$ barristors, obtained by varying $V_{\mathrm{BG}}$, respectively. The application of $V_{\mathrm{BG}}$ changed the Schottky barrier height between the graphene and the semiconductor (CNT or MoS${}_{2}$) by adjusting the Fermi energy of the semiconductor, and shifted the threshold voltages in the transfer curves for each barristor. At the same time, the application of $V_{\mathrm{BG}}$ changed the resistance of the semiconductor itself by electrostatic doping. The total resistance of the barristor is the sum of the resistance of Gr, Gr/semiconductor junction, and the semiconductor, $R_{\mathrm{total}}=R_{\mathrm{Gr}}+R_{\mathrm{junction}}+R_{\mathrm{semi}}$, where $R_{\mathrm{Gr}}$ is negligible due to the semimetallic nature of the graphene. Therefore, a negative $V_{\mathrm{BG}}$, which decreased the resistance of CNT by hole doping, resulted in a higher on-current, on/off ratio, effective mobility and reduced SS [21]. Note for the Gr/MoS${}_{2}$ barristor, a negative $V_{\mathrm{BG}}$ worked in the opposite way in Figure 4b, increasing the resistance of MoS${}_{2}$. Figure 4c displays the subthreshold swing of each barristor, SS = *d*($V_{\mathrm{TG}}$)/*d*(log$I_{\mathrm{DS}}$), as a function of $V_{\mathrm{BG}}$. SS increased with increasing $V_{\mathrm{BG}}$ for the p-type Gr/CNT barristor, and decreased for the n-type Gr/MoS${}_{2}$ barristor, reflecting the change of $R_{\mathrm{semi}}$. SS of each barristor crossed each other at $V_{\mathrm{BG}}≃$−20 V with the value of SS ≃ 160 mV/dec. The larger gain of the complementary inverter observed between –30 V $\leq V_{\mathrm{BG}}\leq$ –15 V (Figure 3b) can be attributed to the fact that both p-and n-type barristors have as small SS as possible in the range of $V_{\mathrm{BG}}$. On the other hand, the dependence of the power consumption on $V_{\mathrm{BG}}$ can be associated with the threshold voltage of each barristor. The power consumption of a complementary inverter is expected to be lower if each component is located more in the deep subthreshold region at $V_{M}$. Therefore, adjustment of the threshold voltages, $V_{\mathrm{Tp}}$ and $V_{\mathrm{Tn}}$, for p-and n-components, respectively, is preferred for the optimization of the inverter. The application of $V_{\mathrm{BG}}$ does not allow individual control over $V_{\mathrm{Tp}}$ and $V_{\mathrm{Tn}}$ and shifts both in the same direction. However, the degrees of change in $E_{F}$ with respect to $V_{\mathrm{BG}}$ are different for the CNT and the MoS${}_{2}$, and the application of $V_{\mathrm{BG}}$ can alter the relative distance between threshold voltages, $V_{\mathrm{Tn}}-V_{\mathrm{Tp}}$. Denoted in the upper axis of Figure 4c, $V_{\mathrm{Tn}}-V_{\mathrm{Tp}}$ is reduced from 2.6 V to 1.5 V upon the decrease of $V_{\mathrm{BG}}$ from 0 to –30 V. The adjustment of $V_{\mathrm{Tn}}-V_{\mathrm{Tp}}$ enabled the transition of the inverter to occur at lower current for $V_{\mathrm{BG}}=-30$ V, reducing the power consumption of the complementary inverter down to ~0.2 nW. In Figure 4d, the transfer curves of the Gr/CNT and the Gr/MoS${}_{2}$ barristors are displayed together for selected $V_{\mathrm{BG}}$. Notice that the intersection of the transfer curves occurred at a lower current regime for $V_{\mathrm{BG}}=-30$ V. Figure 4e presents effective field-effect mobility of each barristor, extracted from the transfer curves shown in Figure 4a,b, and their dependence on $V_{\mathrm{BG}}$. The mobility of the Gr/CNT barristor was calculated according to the 1D mobility equation, $\mu_{1D}=\frac{L}{c_{g}\;V_{\mathrm{DS}}}\frac{dI_{\mathrm{DS}}}{dV_{\mathrm{TG}}}$, with *L* being the length of the junction and $c_{g}$ being the capacitance per unit length. The mobility of Gr/MoS${}_{2}$ was estimated from the 2D mobility equation, $\mu_{2D}=\frac{L}{W\;c_{g}\;V_{\mathrm{DS}}}\frac{dI_{\mathrm{DS}}}{dV_{\mathrm{TG}}}$, with *W* being the width of the junction and $c_{g}$ being the capacitance per unit area. When varying $V_{\mathrm{BG}}$ from –30 V to +10 V, the effective mobility of the Gr/CNT barristor decreased from ~1300 to ~400 cm${}^{2}$/Vs, while μ for the Gr/MoS${}_{2}$ barristor increased from ~0.4 to ~4.7 cm${}^{2}$/Vs. This behavior in the effective mobility is explained by the increase in the resistance of CNT (MoS${}_{2}$) at a positive (negative) $V_{\mathrm{BG}}$, making the switching of $R_{\mathrm{junction}}$ less significant. Here, we find the mobility of Gr/MoS${}_{2}$ is much lower than Gr/CNT barristor, but the mobility of Gr/MoS${}_{2}$ can be improved up to ~100 cm${}^{2}$/Vs by reducing charged impurities [34,35]. Knowing the mobility and the threshold voltage of each barristor component, one can deduce the threshold of the complementary inverter with the following equation [36],

(1) $$V_{M}=\frac{V_{\mathrm{Tn}}+\sqrt{\frac{k_{p}}{k_{n}}}\left(V_{\mathrm{DD}}+V_{\mathrm{Tp}}\right)}{1+\sqrt{\frac{k_{p}}{k_{n}}}},$$

$$k_{n,p}={\left(\frac{W}{L}\right)}_{n,p}\;\mu_{n,p}\;C_{\mathrm{ox}}.$$

Here, $\mu_{n,p}$ is the mobility of either n-or p-barristor, and *C*${}_{\mathrm{ox}}$ is the capacitance of the top-gate insulator per unit area. Using this equation, $V_{M}$ is estimated to be −2.1 V, −2.3 V, −2.6 V, and −3.3 V when $V_{\mathrm{BG}}$ is −30 V, −15 V, −5 V, and 0 V, respectively. These values are in good agreement with the threshold voltage, $V_{M}$, observed in Figure 3a.

Table 1 summarizes previous reports on low-power complementary inverters based on low-dimensional materials. We find several sub-nW low-power complementary inverters realized with CNT- [32] or TMDCs-FETs [30,33]. Inverters using the TMDCs-FETs operated at relatively higher $V_{\mathrm{DD}}$ due to their higher contact resistance than CNT, and the inverter based on CNT-FETs exhibited a lower power consumption of ~0.1 nW. The value is smaller than ~0.2 nW, observed in our inverter made of Gr/CNT and Gr/MoS${}_{2}$ barristors. However, the low-power complementary inverters employing FETs required much lower EOT compared to our device, for example at EOT ~0.7 nm for the inverter realized with CNT-FETs. EOT indicates the thickness of silicon oxide that provides the same electrical performance as that of the dielectric material being used. In principle, thinner gate oxide leads to a smaller SS, reducing the power consumption of the device. Therefore, the low power consumption (~0.2 nW) achieved with a larger EOT of 15 nm for our device represents the excellent switching characteristics of Gr barristors, and further improvement can be expected by lowering the EOT. A theoretical analysis showed that SS of ~50 mV/dec can be achieved in a graphene barristor with EOT ≤1 nm, overcoming the limitation of 60 mV/dec in conventional FETs [34]. Apart from our work, there have been several demonstrations of inverters based on graphene barristors incorporating Si [19], organic semiconductors [31], or cobaltite [37] as p-type semiconductors. However, these p-type Gr barristors showed relatively poor subthreshold swing (SS ≥500 mV/dec) compared to that (SS ≃150 mV/dec) of the p-type Gr/CNT barristor, and limited the performance of the complementary inverter. On the other hand, in order to make a low-power inverter with a high gain, not only SS values of p-and n-components should be low, but transfer curves of p-and n-components should also intersect in the deep subthreshold region [38]. In this research, we relied on $V_{\mathrm{BG}}$ to partly adjust the threshold voltages of each component. A chemical doping of graphene [39], or separate electrostatic doping to each component can allow more precise control over the threshold voltages. In addition, such control over the threshold voltage can make the device to operate at the same input and output voltage ranges, essential for the circuit integration.

Finally, we compare our device with a low-power inverter recently demonstrated using negative capacitance based FETs (NCFETs) by integrating a ferroelectric layer within the gate stack [41]. The complementary inverter with MoS${}_{2}$ and WSe${}_{2}$ NCFETs reported the lowest power consumption of 0.07 nW for the forward sweep and 0.17 nW for the reverse sweep [41]. However, the hysteresis of the inverter, theoretically existing as a result of polarization switching, is a serious obstacle to their practical use. Inverters based on graphene barristors are free from such problem of the ferroelectric negative capacitance and there is much room for the improvement with the reduction of EOT.

## 4. Conclusions

In conclusion, combining a p-type Gr/CNT barristor with a n-type Gr/MoS${}_{2}$ barristor, a complementary inverter was fabricated and its characteristics were investigated. The complementary inverter exhibited a low switching power consumption of ~0.2 nW and a moderate gain of ~2.5 at $V_{\mathrm{DD}}=0.5$ V and at EOT of ~15 nm. This sub-nW complementary inverter was achieved at much larger EOT compared to the inverters based on FETs, which was attributed to the excellent switching characteristics of Gr barristors. The p-type Gr/CNT barristor used in this experiment displayed an on–off current ratio of 10${}^{5}$ and a high mobility of ~1300 cm${}^{2}$/Vs with a subthreshold swing of 150 mV/dec with an EOT of 15 nm. The emergence of a p-type Gr/CNT barristor with an excellent switching property, not available for Gr/TMDCs barristors, opens the possibility of logic devices based on graphene barristors.

## Figures and Tables

**Table 1:** Comparison between low-power complementary inverters.

p-Type	n-Type	$V_{\mathrm{DD}}$ [V]	EOT [nm]	Gain	Power [nW]	Ref
Si	MoS${}_{2}$	1	18	2	2	[29]
MoTe${}_{2}$	MoS${}_{2}$	1	7	2	0.4	[30]
CNT	CNT	0.2	0.7	9	0.1	[32]
CNT	MoS${}_{2}$	5	100	1.3	–	[40]
WSe${}_{2}$	WS${}_{2}$	1.5	2.5	40	1	[33]
Gr/Si	Gr/Si	2	–	1.2	–	[19]
Gr/DNTT	Gr/ZnO:N	2	6.5	8	>10	[31]
Gr/BSCO	Gr/MoS${}_{2}$	2	10	1.6	–	[37]
Gr/CNT	Gr/MoS${}_{2}$	0.5	15	2.5	0.2	This work

## Data Availability

The data is included in the main text.
